# Differential Regulation of Gene Expression, Ion Homeostasis, and Antioxidant Defense Confers Salinity Tolerance During Seed Germination in Wheat

**DOI:** 10.3390/plants15020230

**Published:** 2026-01-12

**Authors:** Ahmed Sallam, Nouran M. Hasseb, Mohamed A. Karam, Andreas Börner, Xu Zheng, Yasser S. Moursi

**Affiliations:** 1 Faculty of Biotechnology, Badr University in Assiut (BUA), Assiut 71531, Egypt; amsallam@aun.edu.eg; 2 Leibniz Institute of Plant Genetics and Crop Plant Research (IPK), D-06466 Gatersleben, Germany; boerner@ipk-gatersleben.de; 3 Department of Genetics, Faculty of Agriculture, Assiut University, Assiut 71526, Egypt; 4 Department of Botany, Faculty of Science, Fayoum University, Fayoum 63514, Egyptmak04@fayoum.edu.eg (M.A.K.); 5 Center for Lipid Engineering, Muyuan Laboratory, 110 Shangding Road, Zhengzhou 450016, China

**Keywords:** wheat, salinity, gene expression, *AVP1*, *NHX1*, ion homeostasis, antioxidants

## Abstract

Salinity represents a major constraint on plant development and crop productivity in wheat, which represents one of the most critical sources of dietary calories worldwide. Its detrimental effects are particularly pronounced during the early stages of growth, including seed germination and seedling establishment. Salinity tolerance is a multifaceted trait governed by several interrelated mechanisms, notably ion homeostasis, osmotic adjustment, activation of enzymatic antioxidant systems, and transcriptional regulation of ion transporter genes. In the present study, contrasting wheat genotypes exhibiting differential salinity tolerance were selected from a panel of 172 accessions evaluated under salinity stress (175 mM NaCl) and control conditions (0 mM NaCl). The objectives of the current study are to confirm the underlying physiological and molecular mechanisms conferring salinity tolerance. Key physiological and molecular parameters including Na^+^, K^+^, and P homeostasis; activities of major antioxidant enzymes; and expression profiles of the salinity-responsive ion transporter genes *TaAVP1* and *NHX1* were quantified in six tolerant genotypes and one susceptible genotype. The tolerant genotypes exhibited higher concentrations of Na^+^ and K^+^ and elevated activities of all antioxidant enzymes, compared with the susceptible genotype. Furthermore, the tolerant genotypes showed differential expression of *TaAVP1* and *NHX1*: both genes were upregulated in Javelin 48 and Kandahar, whereas they were downregulated in genotype 1018d. Notably, genotype Kule demonstrated the highest Na^+^ accumulation, accompanied by markedly elevated activities of all major antioxidant enzymes, with ascorbate peroxidase and glutathione reductase increasing by 9.20-fold and 2.32-fold, respectively, under salinity stress. Based on these findings, the tolerant genotypes can be categorized into two functional groups: Javelin 48, Ghati, and 1018d (characterized by high K^+^ and salinity tolerance) are better suited to soils affected by low Na^+^ salinity, whereas Kandahar, Kule, and 1049 (characterized by high Na^+^ and sodicity tolerance) are more adapted to soils with elevated Na^+^ levels. In conclusion, the tolerant genotypes exhibited distinct, coordinated mechanisms to mitigate salinity stress, underscoring the complexity and plasticity of adaptive responses in wheat.

## 1. Introduction

Wheat is one of the most important staple foods in the human diet; however, its production is increasingly threatened by climate change. Salinity represents a significant challenge to plant growth and crop productivity. Among the various stages of the plant life cycle, seed germination and early seedling development are the most susceptible to salinity stress. High salinity can delay the onset of germination, slow its progress, and increase variability in germination timing, ultimately leading to reduced plant growth and lower final yields [1,2,3,]. Plant species show a broad spectrum of salinity tolerance, as evidenced by their capacity to grow in environments ranging from freshwater to highly saline habitats. Among crop plants, there is significant variation in salt tolerance, not only between different species but also within the same species. These observations highlight two important points: (1) crop plants possess the potential to adapt to saline conditions, and (2) intraspecific variation provides valuable sources to investigate the mechanisms underlying salt tolerance and sensitivity [4,5,6]. Ionic imbalance and nutrient deficiency are among the consequences of salinity stress [7]. The effects of salinity on the nutrient composition of plant tissues have been extensively investigated. Evidence from numerous studies indicates that the detrimental impact of salinity on plant growth is primarily associated with disturbances in ionic homeostasis. In particular, imbalances involving essential cations such as calcium (Ca^2+^) and potassium (K^+^) are recognized as critical factors that disrupt cellular processes, including membrane stability, enzymatic activity, and osmotic regulation, ultimately impairing overall plant performance under saline conditions [8]. The accumulation of high levels of ions is more costly than the assimilation of carbon in the form of organic solutes [9,10,11]. During seed germination and the establishment of young seedlings, the mineral nutrient reserves stored within the seed must be adequate to sustain growth until the seedling achieves physiological self-sufficiency [12]. This indicates the importance of nutrient availability in supporting metabolic and structural development during the very early stages of plant growth under both optimal and stressful conditions. Potassium is an important macronutrient that can account for up to 10% of the cellular dry matter and plays a fundamental role in maintaining the water status of the plant, stomatal movement, enzyme activity, osmoregulation, and membrane stability [13,14,15]. Maintaining high K^+^/Na^+^ ratios is considered among the selection criteria for salt tolerance [16]. Thus, plants must maintain a relatively high concentration of potassium if they are to grow in a saline soil environment [17]. However, sodium is not classified as a macro nutrient because it is a nonessential element for most plants [18]. Under salinity stress, potassium competes for plant uptake through high-affinity potassium transporters and nonselective cation channels, resulting in membrane depolarization, and plants cannot discriminate between Na^+^ and K^+^ ions [19,20]. Sodium is known to cause the most deleterious effect of salinity, and its exclusion is effective for conferring salinity tolerance. The sequestration of high levels of Na^+^ confers salinity tolerance in barley, and Na^+^ might act as an osmolyte or a nutrient to compensate for K^+^ deficiency [21]. Similar results have been reported in other cereals, such as rice [22] and maize [23] where high levels of Na^+^ in the cell vacuole can increase salinity tolerance [24,25]. Phosphorus (P) is an important macronutrient for plant growth and development and plays crucial roles in energy production, DNA synthesis, and salinity tolerance during seed germination [26]. The phosphorus content decreased in seedlings of cotton, wheat, barley, and faba bean under salinity stress [27].

However, balanced production and scavenging of reactive oxygen species (ROS) are highly important for seed germination, suggesting that seed germination and ROS production are coupled [28,29]. Many studies have reported the induction and accumulation of high levels of both ROS and plant antioxidants in relation to salt stress. High levels of ROS can disturb normal metabolism by oxidizing lipids, proteins, and nucleic acids if not detoxified by protective antioxidant systems. It is now widely accepted that ROS are responsible for various types of stress-induced damage to macromolecules and ultimately to the cell [30,31,32]. Plants have a well-developed complex antioxidant system that includes enzymatic and nonenzymatic antioxidant processes to intercept ROS [33]. The antioxidant enzyme network includes many enzymes, among them superoxide dismutase (SOD), catalase (CAT), peroxidase (POD), and ascorbate peroxidase (APX), which detoxify H_2_O_2_ [34]. The activity of all these antioxidant enzymes increases under salinity stress during seed germination and seedling establishment in wheat, maize, and barley coupling with the overproduction of ROS [35,36,37]. The response of plants to environmental cues is under polygenic control. Exploring the connection between gene expression and physiological mechanisms remains a gab in selection for improving plant tolerance to these cues [38]. Approximately 19% of wheat genes are salt-responsive [39]. Salt tolerance in wheat has been suggested to be regulated primarily by the activation of transporter genes [40]. Among them are the vacuolar pyrophosphatase (*V-PPase*) and the Na^+^/H^+^ antiporter. *TaAVP1* expression was equivalent to that of vacuolar Na^+^/H^+^ antiporters in several plant species, indicating that *TaAVP1* generates a proton gradient that is essential for energizing *TNHX1*, which in turn controls vacuolar Na^+^ sequestration, as reviewed by Mansour [41]. Both *V-PPase* and tonoplast Na^+^/H^+^ antiporters are more active in salt-tolerant genotypes than in susceptible genotypes in both rice and citrus [42,43]. The overexpression of Arabidopsis *V-PPase* improved salinity tolerance in alfalfa, and the resulting transgenic plants accumulated high levels of Na^+^, K^+^, and Ca^2+^ ions [44]. Compared with control plants, transgenic plants of finger millet overexpressing *sbVPPase* from sorghum presented higher antioxidant levels and accumulated more Na^+^, K^+^, and Ca^2+^ under 100 mM NaCl and 200 mM NaCl stress [45]. In two contrasting wheat genotypes with respect to salinity tolerance, the expression levels of *TVP1* were similar to those of the vacuolar Na^+^/H^+^ antiporter *TNHX1* in different tissues of the two genotypes [46]. In barley, *HVP10* was upregulated under salinity stress, and transgenic rice plants overexpressing *HVP10* sequestered more Na^+^ in vacuoles and, in turn, exhibited greater salt tolerance than wild-type plants [47].

Although wheat genotypes exhibit considerable intraspecific variability under saline conditions. The physiological and molecular basis for their differential adaptation particularly the classification of tolerant genotypes into salinity-tolerant (low Na^+^ accumulation, high K^+^ retention) and sodicity-tolerant (high Na^+^ sequestration) remains poorly understood. This limits the strategic utilization of genotype-specific mechanisms for targeted breeding in diverse salt-affected soils.

The current study aims to understand the intraspecific variation in the salinity response to study the mechanisms of salt tolerance and sensitivity in wheat. The objectives of the present study were to (1) investigate the molecular basis of salinity tolerance by analyzing the expression patterns of key ion transporter genes (*TaAVP1* and *NHX1*) in tolerant and susceptible genotypes to confirm their contributions to ion homeostasis, (2) evaluate the physiological responses associated with salinity tolerance during early development in contrasting wheat genotypes, focusing on ion homeostasis (Na^+^/K^+^ homeostasis) and antioxidant enzyme activity under salt stress, and (3) confirm the role of intraspecific variation in salinity tolerance and sensitivity.

## 2. Results

### 2.1. Sodium (Na^+^), Potassium (K^+^) and Phosphorus (P)-Related Traits

Ion contents (Na^+^, K^+^ and P) and the ratios K^+^/Na^+^, Na^+^/K^+^ were measured for 7 contrasting wheat genotypes: one susceptible genotype (Sohag-5 from Egypt) and six tolerant genotypes. The details for these genotypes, including gene bank ID, country of origin, and the analyses in which they were involved, are presented in Table 1.

**Figure 1:**
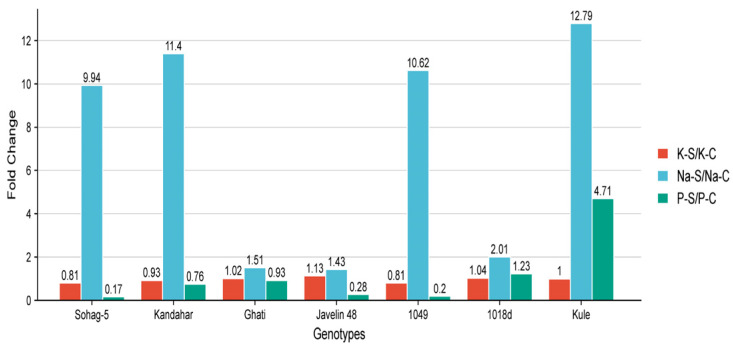
The fold change in Na^+^, K^+^ and P ions in 7 contrasting wheat genotypes under control (0 mM NaCl and salt stress (175 mM NaCl). Na = Sodium, K = Potassium, C = Control and S = Salinity.

**Figure 2:**
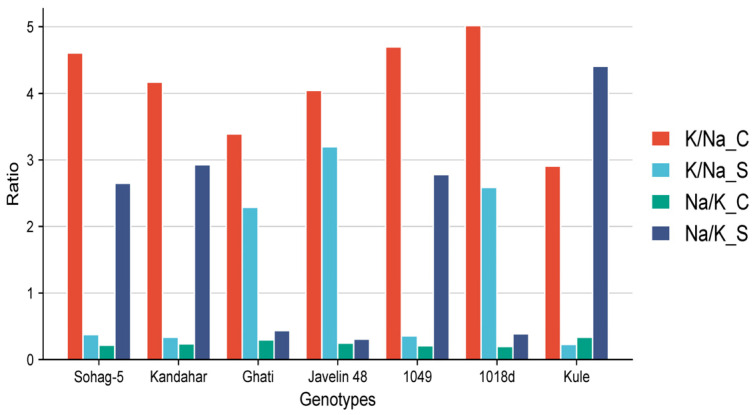
The ratio of Na^+^ and K^+^ ions in 7 contrasting wheat genotypes under control (0 mM NaCl and salinity (175 mM NaCl). K/Na-C = Potassium/Sodium ratio under control, K/Na-S = Potassium/Sodium ratio under salinity, Na/K-C = Sodium/Potassium ratio under control and Na/K-S = Sodium/Potassium ratio under salinity.

All the genotypes showed an increase in Na^+^ content under salinity stress (175 mM NaCl) compared with the control (0 mM NaCl). Among the tolerant genotypes, Javelin 48 from Australia, Ghati from Algeria and 1018d from Morocco maintained the lowest concentrations (Figure S1). The fold change in Na^+^ content under salinity, relative to the control (Na-S/Na-C) was highly pronounced in the susceptible genotype Sohag-5 from Egypt, with a 9.94-fold increase. Similarly, three tolerant genotypes, Kandahar from Afghanistan, 1049 from Morocco, and Kule from Oman, showed substantial fold changes of 11.40, 10.62, and 12.79, respectively (Figure 1). Moreover, the tolerant genotypes varied in Na^+^ accumulation. Among them, the genotypes Javelin 48 from Australia and Ghati from Algeria maintained the lowest levels of Na^+^ under salinity stress, with fold changes of 1.43 and 1.51, respectively (Figure 1). The K^+^ content decreased across all genotypes except Javelin 48, Ghati and 1018d, while remaining unchanged in Kule (Figure 1). As expected, under control conditions, the K^+^/Na^+^ (K/Na-C) ratio was high across all genotypes, ranging from 2.91 in Kule to 5.02 in 1018d. The susceptible genotype Sohag-5 had a ratio of 4.61 (Figure 2). Under salinity, the K^+^/Na^+^ (K/Na-S) ratio decreased in all genotypes; in the tolerant group, it ranged from 0.23 in Kule to 3.20 in Javelin 48, whereas the susceptible genotype Sohag-5 recorded a ratio of 0.38 (Figure 2).

The Na^+^/K^+^ (Na/K-S) ratio was very low in all genotypes under control conditions, ranging from 0.20 for 1018d to 0.30 for Ghati, while the susceptible genotype Sohag-5 had a ratio of 0.22. Under salinity stress, genotypes displayed diverse responses; the ratio increased markedly, reaching 0.31, 0.39 and 0.44 for Javelin 48, 1018d and Ghati, respectively. The susceptible genotype Sohag-5 recorded a substantially higher ratio of 2.65. Regarding phosphorus (P) content, all genotypes showed a pronounced reduction under salinity, except for the tolerant genotypes 1018d and Kule (Figure 2).

### 2.2. The Activity of Some Antioxidant Enzymes Under Control Conditions and Salt Stress

**Figure 3:**
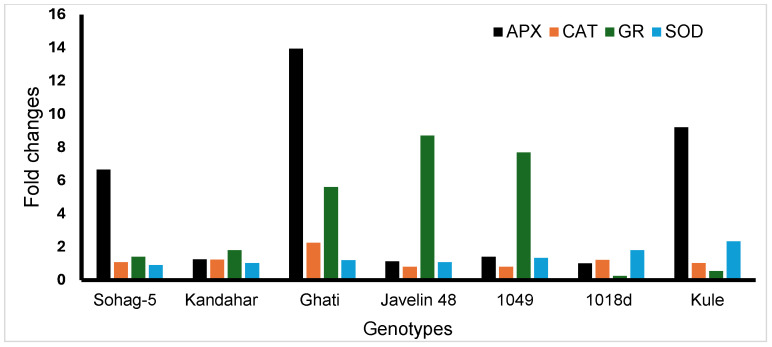
The fold changes in antioxidants enzymes; ascorbate peroxidase (APX), catalase (CAT), glutathione reductase (GR), superoxide dismutase (SOD) in 7 contrasting wheat genotypes under control (0 mM NaCl and salinity (175 mM NaCl).

The activities of the antioxidant enzymes superoxide dismutase (SOD), catalase (CAT), glutathione reductase (GR), and ascorbate peroxidase (APX) were quantified in 7 contrasting wheat genotypes under two treatments: control (0 mM NaCl) and salinity (175 mM NaCl). The enzyme activities varied significantly among the susceptible and tolerant genotypes for all enzymes (Figure S2). Compared with the control, APX activity increased under salinity stress across all seven genotypes. The susceptible genotype Sohag-5 exhibited a 6.65-fold increase, whereas among the tolerant genotypes, Ghati showed the highest increase (13.95-fold), followed by Kule (9.20-fold), while 1018d, showed the lowest fold change (Figure 3). Catalase activity increased under salinity in Sohag-5, Kandahar, Ghati, 1018d, and Kule, meanwhile it decreased in Javelin 48 and 1049 (Figure S2). The greatest increase in CAT activity was recorded in Ghati with 2.25-fold change (Figure 3). SOD activity decreased in Sohag-5, whereas it increased in all of the tolerant genotypes (Figure S2). The greatest increase was recorded in Kule (2.32-fold change), and the lowest increase was in Kandahar, with a 1.03-fold change (Figure 3). GR activity increased under salinity in all genotypes except 1018d and Kule (Figure S2). Among the tolerant genotypes, Javelin 48 exhibited the greatest increase (8.70-fold), whereas Kandahar presented the lowest (1.79-fold). The susceptible genotype Sohag-5 showed a 1.39-fold increase in GR activity (Figure 3).

#### 2.2.1. Analysis of Variance (ANOVA)

The analysis of variance for all measured traits revealed highly significant genotypic (G) and Genotype × environment (G × T) variations (*p* ≤ 0.01), indicating substantial genetic variability among the tested wheat genotypes (Table 2). These findings underscored the genetic variability observed among the genotypes in terms of ionic homeostasis, ion-related traits, and key enzymatic antioxidants. Such variability can be exploited to select and develop genotypes with superior ionic homeostasis and enhanced antioxidant responses under salinity stress. Significant treatment effects were detected for most traits, with the strongest effect observed for K^+^/Na^+^ (*p* ≤ 0.01) and for Na^+^, Na^+^/K^+^, SOD, and APX (*p ≤* 0.05 or 0.1) (Table 2). In contrast, K^+^, CAT and GR were not significantly affected by salinity treatment. The replication effect was mostly non-significant, except for that of Na^+^ and CAT. Oppositely, the (G × T) interaction was significant for all traits (*p* ≤ 0.01), indicating the importance of testing these genotypes under various environmental conditions to select the most environmentally stable genotypes.

#### 2.2.2. Correlation Analysis

**Figure 4:**
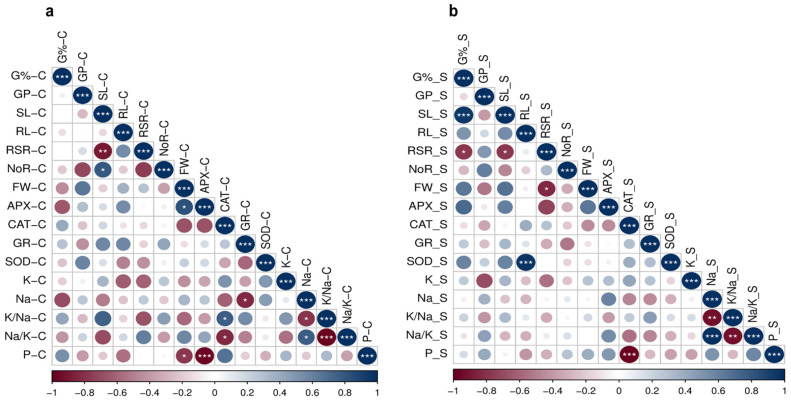
Correlation of morphological traits, antioxidants enzymes, Na^+^, K^+^-related traits and P in 7 contrasting wheat genotypes; (**a**) under control (0 mM NaCl) and (**b**) salt stress (175 mM NaCl). C stands for Control and S stands for Salinity, Germination Percentage (G%), Germination Pace (GP), Shoot length (SL), Root length (RL), Root/Shoot ratio (RSR), Fresh Weight (FW), ascorbate peroxidase (APX), catalase (CAT), glutathione reductase (GR), superoxide dismutase (SOD), Potassium (K), Sodium (Na), Potassium/Sodium ratio (K/Na), Sodium/Potassium ratio (Na/K), Phosphorus (P). *, **, *** represent significance at *p* ≤ 0.05, 0.01 and 0.001, respectively*.*

Under control conditions, the observed correlations ranged from low to highly significant (Figure 4a,b). Positive and significant associations were detected between APX-C and FW-C (r = 0.82 *), CAT-C and K/Na-C (r = 0.77 *), and Na-C and Na/K-C (r = 0.77 *). Conversely, negative and significant correlations were observed between Na-C and GR-C (r = −0.83 *), K/Na-C and Na-C (r = −0.80 *), and Na/K-C with both K/Na-C and CAT-C (r = −0.99 *** and −0.83 *, respectively). Unexpectedly, P-C exhibited a strong negative correlation with APX-C and FW-C (r = −0.99 *** and −0.76 *, respectively). No significant correlations were detected among the enzyme-related traits (Figure 4a).

**Figure 5:**
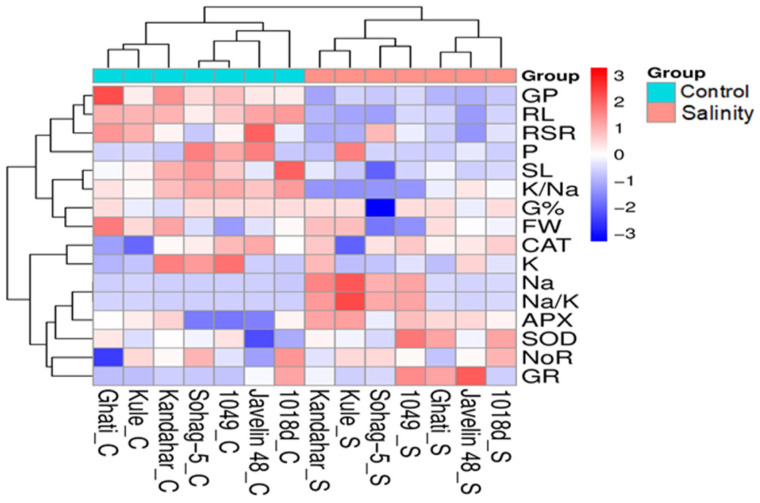
Clustering heat map for the traits of 7 contrasting wheat genotypes; under control (0 mM NaCl) and salt stress (175 mM NaCl). C stands for control and S stands for salinity, Germination Percentage (G%), Germination Pace (GP), Shoot length (SL), Root length (RL), Root/Shoot ratio (RSR), Fresh Weight (FW), ascorbate peroxidase (APX), catalase (CAT), glutathione reductase (GR), superoxide dismutase (SOD), Potassium (K), Sodium (Na), Potassium/Sodium ratio (K/Na), Sodium/Potassium ratio (Na/K), Phosphorus (P).

Under salinity, the correlations ranged from high to very high for the negative significance and were high for positive significance (Figure 5b). Strong positive correlations were detected between SOD-S and RL-S (r = 0.93 ***), indicating that enhanced SOD activity may contribute to root elongation under stress. Similarly, Na/K-S and Na-S showed very high and positive significant correlation (r = 0.99 ***). Expectedly, very high negative significant correlation was observed between K/Na-S and Na/K-S (r = −0.99 **). Also, under salinity, no significant correlations were detected among the enzymatic activity-related traits (Figure 4b). These correlation matrices imply that antioxidant responses operate independently of each other under both conditions control and high salinity.

#### 2.2.3. Bidirectional Clustering Heatmap for All Traits Under Control and Salinity

At the first level of clustering, the genotypes were clustered based on their performance under control and salinity conditions into two primary clusters. The first cluster included the genotypes under control conditions, whereas the second cluster included the genotypes under saline conditions. Within the first cluster, two subclusters were identified: the first contained three tolerant genotypes, with 1018d forming a distinct subgroup, while Javelin 48 and Ghati clustered together. The second subcluster comprised the remaining four genotypes, where 1049 (tolerant) and Sohag-5 (susceptible) were grouped together, and Kandahar and Kule formed a separate pair. Similarly, the second primary cluster, representing genotypes under salinity stress, was further subdivided into two subclusters that mirrored the pattern observed under control conditions (Figure 5).

At the second level of clustering, traits were grouped into two major clusters. The first cluster comprised all morphological traits except the number of roots (NoR) and included two physiological traits: phosphorus (P) content and the potassium-to-sodium ratio (K^+^/Na^+^). This cluster was further subdivided into three subclusters: the first contained P content; the second included the root-to-shoot ratio (RSR); and the third comprised germination pace (GP) which refers to the rate at which seeds complete germination over time and root length (RL). These hierarchical groupings suggest that morphological and certain physiological traits exhibit distinct clustering patterns, reflecting their differential roles in salinity tolerance. Similarly, the second cluster was subdivided into two primary subclusters. The first included fresh weight (FW) and germination percentage (G%), while the second comprised shoot length (SL) and the (K^+^/Na^+^). The second major cluster encompassed traits related to enzymatic activity and ion homeostasis, including Na^+^, K^+^, and P. This cluster was further divided into two subclusters: the first grouped CAT with K^+^ content, whereas the second was partitioned into four smaller subclusters. These included: Na^+^ and the Na^+^/K^+^ in subcluster one, APX in subcluster two, SOD activity in subcluster three, while NoR and GR grouped together in subcluster four. This hierarchical organization underscores the distinct clustering patterns between morphological traits, physiological parameters, and antioxidant enzyme activities, highlighting their differential contributions to salinity tolerance mechanisms.

#### 2.2.4. Principal Component Analysis (PCA) for All Traits Under Control and Salinity

**Figure 6:**
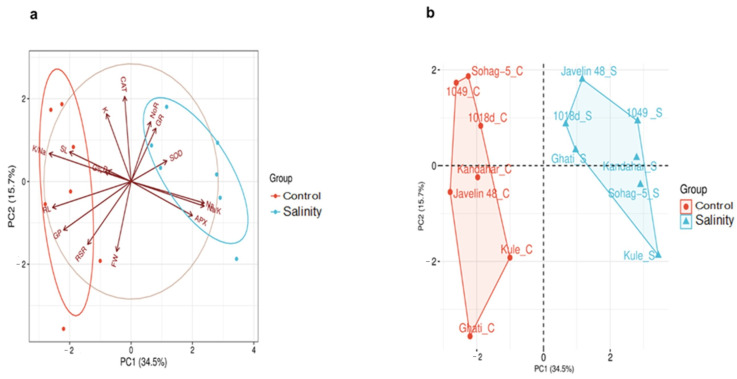
Principal Component Analysis (PCA) in 7 contrasting wheat genotypes under control (0 mM NaCl and salinity (175 mM NaCl), (**a**) biplot of the traits, and (**b**) PCA shows the clustering of genotypes. Germination Percentage (G%), Germination Pace (GP), Shoot length (SL), Root length (RL), Root/Shoot ratio (RSR), Fresh Weight (FW), ascorbate peroxidase (APX), catalase (CAT), glutathione reductase (GR), superoxide dismutase (SOD), Potassium (K), Sodium (Na), Potassium/Sodium ratio (K/Na), Sodium/Potassium ratio (Na/K), and Phosphorus (P).

In the biplot, at the trait level, principal component 1 (PC1) and principal component 2 (PC2) explained 34.5% and 15.7% of the total variation, respectively (Figure 6a). The traits were grouped into two main groups. The first group (red group) comprised traits that accounted for the greatest proportion of variation under the control treatment, whereas the second group (blue group) included the traits contributing most to variation under salinity. The opposite directions of the arrows reflect the correlations between traits; for example, K^+^/Na^+^ and Na^+^/K^+^ exhibited the strongest negative correlation (r = −1.00 ***). The length of each arrow indicates the contribution of the corresponding trait to the variation among the genotypes under a given treatment. For instance, K^+^/Na^+^ and RL are the most discriminative factors under control, whereas Na^+^ and Na^+^/K^+^ were the most discriminative factors among the genotypes under salinity (Figure 6a).

At the genotype level, PC1 and PC2 explained 34.5% and 15.7% of the variation, respectively (Figure 6b). The genotypes were separated into two distinct groups. The first group (red group) shows the performance of the genotypes under the control, and the second group (blue group) shows the performance of the genotypes under salinity. Notably, genotypes such as Kule, Javelin 48, and Ghati exhibited strong (genotype × treatment) interactions, occupying distinct positions across both PC1 and PC2 (Figure 6b). The susceptible genotype Sohag-5 showed relatively modest (genotype × treatment) interactions, with only minor shifts across both PC1 and PC2 axes (Figure 6b). These patterns indicate that tolerant genotypes possess greater phenotypic plasticity under salinity stress compared to the susceptible genotype, which maintained a narrow adaptive response.

### 2.3. Gene Expression Profiling of the Vacuolar Pyrophosphatase (*TaAVP1*) Gene and the Na^+^/H^+^ Antiporter (NHX1) Under Control and Salt Stress

The expression patterns of both the Vacuolar pyro phosphatase gene (TaAVP1), and the Na^+^/H^+^ antiporter (*NHX1*) gene have previously been reported in salt-tolerant Chinese wheat [48]. Among the seven contrasting genotypes that were evaluated for the Na^+^ and K^+^ contents and antioxidant enzyme activities, four contrasting genotypes, including the susceptible genotype; Sohag-5, and three tolerant genotypes Javelin 48, Kandahar, and 1018d were selected for quantifying the gene expression profiles of both genes under control and salinity conditions. The remaining three genotypes Kule, Ghati and 1049 were excluded from the gene expression analysis due to poor RNA quality.

**Figure 7:**
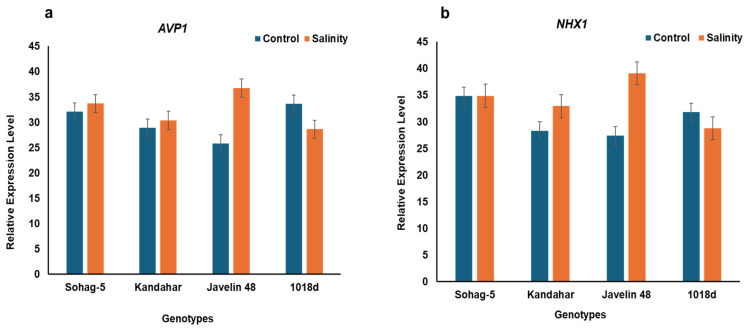
Relative expression levels of (**a**) *APV1* gene and (**b**) *NHX1* gene in four contrasting wheat genotypes; one susceptible genotype “Sohag-5” and three tolerant genotypes “Kandahar, Javelin 48, and 1018d”, under control (0 mM NaCl) and salinity (175 mM NaCl).

Under salinity relative to the control, both genes showed low to moderate changes in expression (Figure S3). The two genes presented different expression patterns in the susceptible genotype (Sohag-5); *TaAVP1* was slightly upregulated, showing a 1.05-fold change, whereas *NHX1* remained changed. The tolerant genotypes differentially expressed both the *TaAVP1* and *NHX1* genes. The *TaAVP1* gene *was* most strongly upregulated in Javelin 48 (1.43-fold), followed by Kandahar (1.05-fold), while in 1018d it was downregulated (Figure 7a and Figure S3). Similarly, *NHX1* showed genotype-specific responses: Javelin 48 exhibited the highest upregulation (1.42-fold), Kandahar showed moderate upregulation (1.16-fold), and 1018d displayed downregulation (Figure 7b and Figure S3). These findings indicate that salinity tolerance in the given wheat genotypes involves genotype-dependent regulation of vacuolar ion transporters, with Javelin 48 and Kandahar activating both genes under stress, whereas 1018d employes a contrasting strategy.

## 3. Discussion

Plants cannot escape abiotic stresses such as salinity; therefore, they have developed various mechanisms to combat its detrimental effects. Salinity induces alterations in all plant parts, extending from individual cells to the whole plant. The response to salinity involves complex adjustments across several pathways, including ion homeostasis, osmotic regulation, activation of antioxidant defense systems, and modulation of genes that govern ion transport through exclusion and vacuolar sequestration. Notably, ion homeostasis via Na^+^ exclusion or vacuolar sequestration represents a fundamental mechanism for conferring salinity tolerance [19].

The genotypes in this study revealed highly significant genotypic variation (*p* ≤ 0.01) for all measured traits, including ion-related traits (K^+^, Na^+^, K^+^/Na^+^, Na^+^/K^+^, P) and antioxidant enzyme activities (SOD, CAT, APX, GR) as confirmed by ANOVA (Table 2). This high genetic diversity highlights the potential for exploiting these traits as reliable selection criteria for improving salinity tolerance in wheat. The observed variability among genotypes further supports the potential for genetic improvement through targeted selection of genotypes with superior ionic balance and enhanced antioxidant responses under salinity stress. In terms of the genotypic response to salinity, Sohag-5 consistently clustered separately from the tolerant genotypes under salinity conditions, confirming its divergent response profile, whereas the tolerant genotypes (especially Kandahar, 1018d, and Kule) grouped closely, reflecting adaptive traits shared under salinity stress (Figure 5). Compared with the susceptible genotype Sohag-5, the tolerant genotypes exhibited greater genotypic plasticity, as evidenced by their distinct shifts on the PCA axes between control and salinity conditions. In contrast, Sohag-5 displayed limited plasticity, maintaining a relatively stable position across treatments, as shown in Figure 6b.

### 3.1. Roles of Mineral-Related Traits and Antioxidant Enzymes in Salinity Tolerance

Our results revealed that the susceptible genotype (Sohag-5) accumulated markedly high levels of Na^+^ under salinity compared with the control. The tolerant genotypes presented different responses and were separated into two groups. The first group included Javelin 48, Ghati and 1018d, which maintained low Na^+^ concentration, low Na^+^/K^+^ ratios, high K^+^ concentrations, and high K^+^/Na^+^ ratios under salinity stress, whereas the second group included Kandahar, Kule and 1049, which maintained high concentrations of Na^+^, low K^+^ concentration, high Na^+^/K^+^ ratios and low K^+^/Na^+^ ratios (Figure S1 and Figure 2). These results indicate that tolerant genotypes employ distinct mechanisms to tolerate salinity. More likely, Javelin 48 and Ghati exclude excess Na^+^ while maintaining Na^+^ and K^+^ levels at physiological concentrations for all biological processes. On the other hand, salt-tolerant Na^+^ accumulators appear to utilize this ion for osmotic adjustment, thereby sustaining water uptake under salinity stress. Our findings revealed that, under salinity the tolerant genotypes; Kandahar, 1049 and Kule did not accumulate high K^+^ concentrations; nevertheless, they remain among the tolerant genotypes that might benefit from the accumulation of excess Na^+^ (Figure S1). Similarly, in Arabidopsis and barely, natural variation in salinity tolerance is achieved by maintaining high K^+^ and K^+^ and K^+^/Na^+^ ratios in the most tolerant genotypes [49,50]. In support of these results, studies in Arabidopsis and wheat at the seedling stage have demonstrated that high K^+^ concentrations sustain plant growth under salinity stress by preserving a high K^+^/Na^+^ ratio, reducing the Na^+^/K^+^ ratio, reducing reactive oxygen species (ROS) production, and enhancing antioxidant enzyme activity [50,51]. In another study in wheat, the tolerant wheat genotype Sakha 93 maintained a high K^+^ content compared with the susceptible genotype Sakha 61 [52]. The retention of high K^+^ under salinity stress confers salinity tolerance in various cereals, including rice [53], wheat [54] and barley [55]. Potassium is a key player in conferring salinity tolerance by tailoring various biological processes [56]. In line with our results, several studies reported that K^+^/Na^+^ is essential for salinity tolerance in bread wheat [57], barley [58], and maize [59]. This high K^+^/Na^+^ ratio not only mitigates ionic imbalance but also supports metabolic stability, thereby enhancing plant growth and resilience in saline environments.

Notably, the tolerant genotypes Kandahar, 1049 and Kule maintained higher Na^+^ concentrations than even the most susceptible genotype, Sohag-5 (Figure S1). Presumably, these genotypes employ alternative mechanisms to achieve salinity tolerance. Compared with the remaining tolerant genotypes, they likely presented more efficient partitioning and localization of excess Na^+^ content. The greatest increases in Na^+^ content under salinity relative to the control were 10.62-, 11.40- and 12.79-fold greater for 1049, Kandahar and Kule, respectively, which also presented the highest Na^+^/K^+^ ratios (Figure 1 and Figure S1). Moreover, Kule maintained the highest P content under salinity, which may contribute in seedling growth or serve as an osmoticum. In support of our findings, salinity- and sodicity-tolerant wheat lines have been shown to retain high levels of Na^+^ [60], indicating that sodium accumulation can serve as an adaptive mechanism for osmotic adjustment under saline conditions. Under salinity stress, K^+^ uptake is reduced; in such cases, Na^+^ serves as a cost-effective osmoticum that can substitute for the biosynthesis of organic osmolytes, a process that is both time- and energy-consuming for plant cells [9,11,61]. Additionally, Na^+^ can compensate for K^+^ deficiency, thereby enabling continuous shoot growth under salinity stress [11]. Our results agree with other previous studies. Ashraf et al. [62] reported that, in wheat, salinity stress increased the Na^+^ content in both salt-tolerant and salt-susceptible genotypes; however, the tolerant genotypes presented high K^+^/Na^+^ ratios compared with the susceptible genotypes. Similarly, six Malawian tomato cultivars were evaluated for salinity tolerance under 200 mM NaCl, and the tolerant cultivar maintained high K^+^ and low Na^+^ concentrations, resulting in a higher K^+^/Na^+^ ratio compared with the susceptible cultivar [63]. Phosphorus availability helps maintain other essential minerals, such as Mg^2+^, and supports Na^+^ exclusion, as reviewed by Khan et al. [64].

The role of enzymatic antioxidants in mitigating salinity stress has been demonstrated in several model and crop plant species. In the present study, all genotypes presented increased antioxidant enzyme activities, with the exception of SOD in Sohag-5, CAT in Javelin 48 and 1049, as well as GR in 1018d and Kule (Figure 3). The increase in SOD was expected, as SOD represents the frontline of a plant’s ROS scavenging system. SOD catalyzes the first step in neutralizing ROS by converting O_2_^−^ to H_2_O_2,_ which remains toxic to the cell; therefore, H_2_O_2_ is subsequently decomposed by CAT into H_2_O and O_2_ [62]. In line with our results, an increase in SOD activity was observed under salinity stress in the wheat-tolerant genotype (Kharchia65) compared with the susceptible genotype (HD2687) [65]. Similarly, in wheat at the seedling stage, compared with the control treatment, exposure to 120 mM NaCl treatment significantly induced the SOD activity [66]. Our results revealed that SOD was highly and positively correlated with RL_S (r = 0.93 ***) (Figure 4b). SOD activity increased in all genotypes except the susceptible genotype (Sohag-5), indicating that enhanced SOD activity plays a critical role in conferring salinity tolerance. Likewise, SOD induced activity improved plant growth under salinity stress in Arabidopsis [67]. The unchanged levels of CAT in the tolerant genotypes are consistent with previous findings in maize under salinity stress [68]. Probably, this is attributed to the inhibitory effect of the oxidative stress or low enzymatic activity [69,70,71]. Additionally, some studies stated that CAT may not be the primary H_2_O_2_ scavenger under salt stress [72].

The APX activity increased in all genotypes, including the susceptible genotype Sohag-5, and the increase was more pronounced in the tolerant genotypes, Ghati and Kuli (Figure 3). These results suggest that APX contributes at conferring salinity tolerance in the tolerant genotypes, even in those exhibiting high Na^+^ concentrations. Notably, the APX activity of Ghati and Kule increased most markedly, by 13.95-fold and 9.20-fold, respectively. APX was found to be positively correlated with plant growth under salinity in Arabidopsis [67]. This may be attributed to the high affinity of APX for H_2_O_2_ in different organelles when ascorbate is used as an electron donor [73]. APX alleviation enhanced the salinity tolerance of wheat under salinity stress induced by 120 mM NaCl [66]. APX markedly increased under 350 mM NaCl treatment in neglected and ancestral wheat relatives at the young plant growth stage, and the expression of the APX gene was highly upregulated [74]. Similar findings regarding APX induced activity and APX gene upregulation have been reported in two contrasting wheat varieties, where the tolerant genotypes exhibited higher APX content and greater APX gene expression compared with the salt-susceptible variety [75].

For GR, a genotype-dependent pattern was observed, and salinity stress increased GR activity in all genotypes except the two tolerant genotypes, Kule and 1018d (Figure 3). The greatest increases were observed in the two tolerant genotypes, 1049 and Javelin 48, with 7.68- and 8.70-fold changes, respectively. Several studies have reported an increase in GR in wheat under different concentrations of salinity stress and at different growth stages [51,62,66,75,76]. In a comparative study between two Egyptian wheat cultivars, the salinity-tolerant cultivar (Misr 2) and salinity-susceptible cultivar (Sakha 95), Misr 2 showed higher GR activity and exhibited greater salinity tolerance than Sakha 95 [77]. The GR enzyme is a key player in plant tolerance to abiotic stresses, especially salinity, as it maintains optimal levels of reduced glutathione, which is essential for ROS homeostasis through the glutathione–ascorbate cycle.

Taken together, the antioxidant enzymes SOD, CAT, APX and GR play a protective role in plant tolerance to salinity by neutralizing the oxidative damage caused by the excessive ROS generated under salinity stress. Our findings revealed significant intraspecific variability in the activity of all enzymes. Salinity stress increased enzymatic activity even in the salt-susceptible genotype Soahg-5; however, for most enzymes, the tolerant genotypes exhibited the highest activity levels (Figure 4a,b and Figure 5).

To sum up, our findings indicate that salinity tolerance in wheat involves various strategies such as ion homeostasis (K^+^ retention, Na^+^ exclusion or sequestration) and activation of antioxidant defense systems, with significant intraspecific variability among genotypes.

### 3.2. Gene Expression Profiling of the AVP (A Vacuolar Pyrophosphatase Similar to AVP1) and NHX1 (A Na^+^/H^+^ Antiporter)

The expression profiles of both genes were estimated in four contrasting genotypes: one salt-susceptible genotype (Sohag-5) and three salt-tolerant genotypes (Javelin 48, Kandahar and 1018d). The two genes exhibited distinct expression profiles under salinity treatment compared with the control. In the salt-susceptible genotype (Sohag-5), *TaAVP* was slightly upregulated (1.05-fold), whereas *NHX1* remained unchanged (Figure 7 and Figure S3). The tolerant genotypes exhibited differential expression of both genes. The highest expression levels of *AVP1* and *NHX1* were observed in Javelin 48 (1.4-fold relative to the control), followed by Kandahar (1.14-fold relative to the control), 1018d exhibited a distinctive pattern, where both genes were downregulated (Figure 7 and Figure S3). These findings indicate that gene expression varied among the susceptible and tolerant genotypes, as well as within the tolerant group, showing a genotype-specific pattern. In the context of low expression levels, our results agree with the findings of Genc et al. [60], who reported low expression levels of both genes in wheat genotypes that accumulated low-Na^+^ concentrations. Our results are consistent with the findings of Nakayama et al. [40], who found that, in tolerant common wheat, salt-responsive genes presented a genotype-specific pattern. Specifically, the Na^+^/H^+^ antiporter gene was significantly upregulated in some salt-tolerant lines, whereas it was downregulated in other tolerant lines. These results indicate that *NHX1* was differentially expressed among the tolerant genotypes. However, the tolerant genotype Javelin 48 exhibited the highest expression levels of both genes, which are likely involved in Na^+^ sequestration within the vacuole. This genotype maintained the lowest Na^+^ content, the highest K^+^ and K^+^/Na^+^ ratio and the lowest Na-S/Na-C fold change among the salt-tolerant genotypes. More likely, it has an efficient Na-excluding active allele of membrane antiporters (*NHX2*), which contributes to maintaining physiological Na^+^ levels. Alternatively, *NHX1* favored the vacuolar sequestration of K^+^ at the expense of Na^+^. In contrast, in Kandahar (a tolerant genotype with high Na^+^ concentrations), Na^+^ appears to be sequestered at the expense of K^+^, reflecting the multifunctional role of *NHX1* in salt-tolerant wheat genotypes. In the present study, both *AVP1* and *NHX1* genes were downregulated in the tolerant genotype 1018d, which exhibited the highest Na^+^ content among the tolerant genotypes. This observation suggests that 1018d uses alternative mechanism to tolerate salinity, such as osmotic adjustment and activation of antioxidant enzymatic systems. This finding is consistent with the report by Gaxiola et al. [78], who reported that the overexpression of *TaAVP1* conferred salinity tolerance in Arabidopsis transgenic plants compared with wild-type plants. The transgenic Arabidopsis plants maintained high levels of both Na^+^ and K^+^ and retained more water. Nakayama et al. [40] found that the *NHX* genes of two salt-tolerant lines were upregulated through different mechanisms, with one mechanism leading to the upregulation of antioxidant genes.

Notably, there was concomitant upregulation/downregulation of both genes in the tolerant genotypes, indicating that both genes are involved in salt tolerance and likely function in a coordinated manner. The simultaneous upregulation of *NHX1* and *AVP1* has been reported in several plant species. *AVP1* expression was equivalent to that of *NHX* antiporters in several plant species, indicating that *AVP1* generates a proton gradient essential for energizing *NHX1,* which, in turn, controls vacuolar Na^+^ sequestration, as reviewed by Mansour [41]. Several studies reported that the coexpression of both the *NHX* and *AVP* genes improves salinity tolerance in transgenic plants expressing both genes simultaneously rather than only one. This improvement has been demonstrated in various plant species, such as rice, sugar beet, lotus, tobacco, and Arabidopsis [79]. In two contrasting wheat genotypes with respect to salinity tolerance, the expression levels of *TaAVP1* were comparable to those of the vacuolar Na^+^/H^+^ antiporter (*TNHX1*) across different tissues of the two genotypes [46].

Our findings demonstrate that salinity tolerance in wheat is linked to the genotype-specific regulation of *TaAVP1* and *NHX1*. Their coordinated upregulation in certain tolerant genotypes suggests a critical role in vacuolar Na^+^ sequestration and proton gradient generation, whereas downregulation in others indicates alternative adaptive strategies. In line with other mechanisms, such as ionic homeostasis and antioxidant enzymes activity, these genes represent promising molecular targets for improving salinity tolerance in wheat.

## 4. Materials and Methods

### 4.1. Plant Material and Experimental Design

The details of the germination experiment and a full description of the plant materials are provided in Hasseb et al. [80]. Briefly, a randomized complete block design (RCBD) with three replicates was used to evaluate a collection of 172 highly diverse wheat genotypes for seed germination and seedling establishment; under two treatments: control conditions (0 mM NaCl) and salinity stress (175 mM NaCl). The data resulting from that experiment were used for genome-wide association analysis (GWAS) [80]. Twenty seeds from each genotype were rinsed with distilled water and surface-sterilized in 1% sodium hypochlorite (NaOCl) for 10 min, followed by three washes with deionized distilled water. The sterilized seeds were placed in Petri dishes containing two layers of Whatman No. 1 filter paper moistened with 10 mL of either control solution (0 mM NaCl) or salinity treatment solution (175 mM NaCl). Petri dishes were incubated at 20 °C in complete darkness. Seeds were considered germinated when the radicle reached 2 mm in length, and germination was recorded every 24 h for 10 days. The germination percentage (G%) and germination pace (GP)) which refers to the rate at which seeds complete germination over time [80,81,82]. For shoot length (SL) and root length (RL) measurements, ten seeds per genotype were grown using the rolling paper method described by Hetz et al. [83]. The rolls were placed vertically in 1 L beakers, half-filled with the corresponding solutions (0 mM NaCl or 175 mM NaCl), which were refreshed every second day to maintain both salinity strength and solution volume. After 12 days, the experiment was terminated, and SL and RL were measured manually in centimeters using a ruler. The root-to-shoot ratio (RSR); was computed as the ration of root length to shoot length.

Based on their seed germination and seedling-related traits values, the 172 genotypes were ranked. From these, seven genotypes, i.e., six (the most tolerant) and one (the most susceptible) were selected for the analyses of ion concentrations, enzymatic antioxidants activities, and gene expression. The selection of six tolerant genotypes and one susceptible genotype was intended to investigate the genotypic plasticity of tolerant genotypes under salinity stress, as these genotypes are of greater value for breeding purposes, whereas the susceptible one was intentional and served as a physiological contrast to validate known salinity-tolerance mechanisms. Three tolerant genotypes Kule, Ghati and 1049 were excluded from the gene expression analysis due to poor RNA quality. All information about the seven genotypes included in this study is presented in Table 1.

### 4.2. Antioxidant Enzyme Activities

#### 4.2.1. Extraction and Measuring

For each genotype, the collected seedling tissue was immediately frozen in liquid nitrogen and stored at −80 °C until further analysis. A frozen seedling tissue (0.3–0.4 g) was finely ground in a pre-chilled mortar and pestle using liquid nitrogen, and the resulting powder was homogenized in 5 mL of 50 mM potassium phosphate (K-P) buffer (pH 7.8) supplemented with 1 mM ascorbic acid. The homogenate was centrifuged at 15,000× *g* for 15 min at 4 °C, and the resulting supernatant was collected and used for subsequent enzyme activity assays. The activities of the antioxidant enzymes superoxide dismutase (SOD, EC 1.15.1.1), ascorbate peroxidase (APX, EC 1.11.1.11), catalase (CAT, EC 1.11.1.6), and glutathione (GR, EC 1.6.4.2) were measured at the corresponding wavelength for each enzyme.

SOD activity was estimated according to Dhindsa et al. [84] by recording the decrease in the absorbance of the superoxide nitro blue tetrazolium (NBT) complex by the enzyme at 560 nm. Approximately 3 mL of the reaction mixture, containing 0.1 mL of 200 mM methionine, 0.1 mL of 2.25 mM (NBT), 0.1 mL of 3 mM (EDTA), 1.5 mL of 100 mM potassium phosphate buffer, 1 mL of distilled water, and 0.05 mL of extracted enzyme extract, was prepared in duplicate tubes for each sample. Additionally, two control tubes were prepared without enzyme extract. The reaction was initiated by adding 0.1 mL of riboflavin (60 μM) to all tubes placed under a light source of two fluorescent lamps (15 W) for 15 min. The reaction was terminated by switching off the light and covering the tubes with dark covers. The maximum color developed in the tubes without the enzyme extract, while a non-irradiated complete reaction mixture that did not produce color was used as a blank.

##### Ascorbate Peroxidase Assay

APX activity was estimated at 290 nm according to Nakano and Asada [85], using spectrophotometric monitoring of the decrease in absorbance as an indicator of the rate of ascorbate oxidation. The reaction mixture consisted of 25 mM phosphate buffer (pH 7.0), 0.1 mM EDTA, 1 mM H_2_O_2_, 0.25 mM ascorbate (AsA) and the enzyme sample.

##### Catalase Assay

The activity of CAT was determined by estimating the rate of H_2_O_2_ decomposition at 240 nm following the method described by Aebi [86]. Approximately 3 mL of the reaction mixture contained 1.5 mL of 100 mM potassium phosphate buffer (pH 7.0), 0.5 mL of 75 mM H_2_O_2_, 0.05 mL of enzyme extract, and distilled water was prepared. The reaction was initiated by the addition of H_2_O_2_, and the decrease in absorbance was recorded for 1 min.

##### Glutathione Reductase Assay

GR activity was estimated in the presence of oxidized glutathione (GSSG) and 5,5′-dithiobis-2-nitrobenzoic acid (DTNB) by recording the increase in absorbance, according to Smith et al. [87]. The reaction mixture consisted of 1 mL of 0.2 M potassium phosphate buffer (pH 7.5) containing 0.1 mM EDTA, 0.5 mL of 3 mM DTNB in 0.01 M potassium phosphate buffer (pH 7.5), 0.1 mL of 2 mM NADPH, 0.1 mL of enzyme extract, and distilled water to make up a final volume of 2.9 mL. The reaction was initiated by adding 0.12 mM GSSG, and the increase in absorbance at 412 nm was recorded at 25 °C over a period of 5 min using a spectrophotometer.

### 4.3. Estimation of Sodium (Na^+^), Potassium (K^+^) and Phosphorus (P) Contents

The samples were dried at 80 °C for 72 h. An electric grinder (Model ECG-200; Hefei Ecocoffee Co., Ltd., Hefei, China, was used to pulverize the dried samples for the determination of Sodium (Na^+^) and Potassium (K^+^) concentrations. One day before digestion, the samples were dried overnight at 100 °C. In a microwave-assisted acid digestion system (Anton Paar Multiwave 5000, Graz, Austria), 0.5 g from each sample was placed in a vessel with 10 mL of nitric acid. At 175 °C, the samples were placed in the microwave system for 10 min (heated to 175 °C in 5.5 min and maintained for 4.5 min). After cooling, the samples were diluted to 50 mL with deionized water. The concentrations of Na^+^ and K^+^ were quantified by atomic absorption spectrophotometry. The K^+^/Na^+^ ratio was calculated based on the measured concentrations of Na^+^ and K^+^. Total inorganic phosphate (Pi) was quantified by adding ammonium vanadomolybdate reagent and measuring the absorbance at 470 nm via a UV/Vis spectrophotometer (T80; PG Instruments Ltd, Leicestershire, United Kingdom). Fold changes in Na^+^, K^+^, and P contents were computed as the ratio of the values under salinity treatment to those under the control condition.

### 4.4. Gene Expression Analysis

Gene expression analysis was conducted on four contrasting genotypes (three tolerant and one susceptible) under both control and salt stress conditions. The remaining three genotypes were excluded from the analysis due to poor RNA quality. The expression levels of two genes were compared among the four genotypes under the two treatments. These two genes, AK4544458/TaAffx.25629.1S1 (vacuolar pyrophosphatase similar to *AVP1*) and AY296910 (Na^+^/H^+^ vacuolar antiporter “*NHX1*”), have been previously reported. The sequences of the forward and reverse primers corresponding to these genes are listed in Table 3.

#### RNA Extraction, cDNA Preparation, and Real-Time PCR

The same NaCl concentrations used in the germination experiment (0 mM NaCl) for the control and (175 mM NaCl) for salinity stress were applied to the four selected genotypes. Three biological samples were collected from the leaves of each genotype (0.1 g per replicate). All leaf samples were immediately transferred to liquid nitrogen and stored at −80 °C until further analysis. RNA was extracted using the RNeasy Plant Mini Kit (Qiagen, Valencia, CA, USA). To obtain purified RNA, all samples were treated with RNase-free DNase I (Thermo Fisher Scientific, Waltham, Massachusetts, USA) Complementary DNA (cDNA) was synthesized using the RevertAid First Strand cDNA Synthesis Kit according to the manufacturer’s protocol (Thermo Fisher Scientific, Waltham, Massachusetts, USA). Real-time PCR was performed using Maxima SYBR Green/ROX qPCR Master Mix (2X) (Thermo Fisher Scientific, Waltham, Massachusetts, USA). The thermocycling conditions were as follows: 2 min at 50 °C, 10 min at 95 °C, followed by 40 cycles of 95 °C for 15 s and 60 °C for 1 min. The expression levels for each sample under both treatments were analyzed in three biological replicates and calculated via the 2^−^^ΔΔCT^ method.

### 4.5. Data Analysis

The analysis of variance (ANOVA) was calculated under both control and salt stress conditions via PLABSTAT software version 3A [88] and the R package, version 4.5.1 [89] via the following statistical model:

*Y_ijk_* = *μ* + *g_i_* + *r_j_* + *t_k_* + *t_ik_* + *tgr_ijk_*

where *μ* is the general mean value; *g_i_*, *r_j_*, *t_k_* are the main effects of genotype, replication, and treatment, respectively. *t_ik_* represents the genotype × treatment interaction. *tgr_ijk_* represents the genotype × replication × treatment interaction (error).

Phenotypic correlation analysis was performed using PLABSTAT. Correlation coefficients were classified as low (0–0.39), moderate (0.40–0.60), and high (>0.60). Data visualization for all the parameters was conducted using SRrplot, a free online platform for data visualization and graphing [90] and Excel 365 [91].

## 5. Conclusions

The current study highlights that wheat salinity tolerance is governed by complex, genotype-specific mechanisms integrating ion homeostasis, ion adjustment, antioxidant defense, and gene expression regulation. Tolerant genotypes display diverse strategies, ranging from Na^+^ exclusion and K^+^ retention to Na^+^ sequestration for osmotic adjustment, supported by high K^+^/Na^+^ ratios and enhanced antioxidant enzyme activities. The simultaneous upregulation of *NHX1* and *TaAVP1* in tolerant genotypes suggests a coordinated role in salinity tolerance. Genotypes such as Javelin 48, Ghati and 1018d (high K^+^, salinity-tolerant) are suitable for salinity-affected soil with low Na^+^, meanwhile Kandahar, Kule and 1049 (high Na^+^, sodic tolerant) are better adapted to soils with high Na^+^ levels. These results highlight the plasticity of adaptive responses and provide valuable molecular and physiological markers for improving wheat salinity tolerance through targeted breeding.

## Figures and Tables

**Table 1:** The Details of genotypes analyzed including gene bank ID, accession name, origin, and analyses of the seven contrasting genotypes (6 tolerant genotypes and 1 susceptible genotype).

Gene Bank ID	Accession Name	Country	Character	Na^+^ and K^+^ Traits	Antioxidant Enzymes Activity	Gene Expression
PI220127	Kandahar	Afghanistan	Tolerant	√	√	√
PI542666	Ghati	Algeria	Tolerant	√	√	×
PI201414	Javelin 48	Australia	Tolerant	√	√	√
PI525241	1049	Morocco	Tolerant	√	√	×
PI525221	1018d	Morocco	Tolerant	√	√	√
PI532249	Kule	Oman	Tolerant	√	√	×
122	Sohag-5	Egypt	Susceptible	√	√	√

(√) stands for included and (×) stands for excluded.

**Table 2:** Analysis of variance (ANOVA) of sodium, potassium and phosphorus traits and antioxidant enzyme activities in 7 contrasting genotypes.

Source of Variance	K^+^	Na^+^	K^+^/Na^+^	Na^+^/K^+^	P	APX	CAT	GR	SOD
Treatments	0.97	8.58 *	26.36 **	8.30 *	0.89	11.26 *	0.92	1.97	5.98 ^+^
Replications	1.47	6.21 **	0.38	3.31 ^+^	0.18	0.11	4.94 *	1.88	2.35
Genotypes	93.93 **	5910.30 **	390.57 **	1151.24 **	9018.13 **	2148.59 **	4565.21 **	392.11 **	89.85 **
Treatment × Genotype	57.25 **	5455.80 **	332.69 **	1098.89 **	12,438.75 **	965.85 **	605.26 **	565.14 **	42.63 **

* and ** represent significance at *p* ≤ 0.05 and 0.01, respectively; + indicates marginal significance (*p* ≤ 0.1).

**Table 3:** Primer sequences used for qPCR analysis.

Accession Number/Probe Set	Annotation/Predicted Function	Primer Sequence (5′-3′)	Amplicon (bp)	Reference
AK4544458/TaAffx.25629.1.S1	Vacuolar pyrophosphatase similar to AVP1 (TaAVP1)	Fwd: GACCGGTCTTGCCATTGATG	162	[43]
		Rev: CTGAGCCAATTGCGAATCCC		
AY296910	Na^+^/H^+^ antiporter (*NHX1*)	Fwd: GCCTGGTTCACCCATAGAGA		
		Rev: CACCGAAAGAATCCCAAGAG	159	[43]

## Data Availability

All relevant data can be found within the manuscript and its supporting materials, and any further inquiries can be directed to the corresponding authors.

## Supplementary Materials

The following supporting information can be downloaded at: https://www.mdpi.com/article/10.3390/plants15020230/s1. Figure S1: The Na^+^, K^+^, and P related traits in 7 contrasting wheat genotypes under control (0 mM NaCl and salt stress (175 mM NaCl). C = control, S = Salinity. Figure S2: Antioxidants enzymes activity; APX = Ascorbate Peroxidase, CAT = Catalase, SOD = Super Oxide Dismutase, and GR = Glutathione Reductase in 7 contrasting wheat genotypes and control (0 mM NaCl and salt stress (175 mM NaCl). Figure S3: The logarithmic fold change in *TaAVP1* and *NHX1* genes in four contrasting wheat genotypes; Sohag-5 “susceptible”, Javelin 48, Kandahar, 1018d “tolerant”.

## Abbreviations

ANOVA	Analysis of variance
APX	Ascorbate peroxidase
ASRT	Academy of Scientific Research and Technology
AVP1	Vacuolar H^+^-pyrophosphatase
CAT	Catalase
cDNA	Complementary DNA
DNase	Deoxyribonuclease
EC	Enzyme Commission number
EDTA	Ethylenediaminetetraacetic acid
FW	Fresh weight
GR	Glutathione reductase
GWAS	Genome-wide association study
H^+^-ATPase	Proton adenosine triphosphatase
H^+^-PPase	Proton pyrophosphatase
H_2_O_2_	Hydrogen peroxide
H_2_PO_4_^−^	Dihydrogen phosphate ion
K^+^	Potassium ion
K^+^/Na^+^	Potassium to sodium ratio
Na^+^	Sodium ion
Na^+^/K^+^	Sodium to potassium ratio
NaCl	Sodium chloride
NaOCl	Sodium hypochlorite
NBT	Nitro blue tetrazolium
NHX1	Na^+^/H^+^ antiporter gene
PCA	Principal component analysis
PCR	Polymerase chain reaction
Pi	Inorganic phosphate
qPCR	Quantitative polymerase chain reaction
RCBD	Randomized complete block design
RL	Root length
RNA	Ribonucleic acid
RNeasy	RNA easy extraction kit
ROS	Reactive oxygen species
SL	Shoot length
SNG	Scientists for Next Generation (Scholarships Program)
SOD	Superoxide dismutase
*TaAVP1*	Vacuolar H^+^-pyrophosphatase gene (in *Triticum aestivum*)
TNHX1	Triticum Na^+^/H^+^ antiporter
TVP1	Triticum vacuolar pyrophosphatase
ΔΔCT	Delta–delta cycle threshold
