# Supplementary material for: Differential Regulation of Gene Expression, Ion Homeostasis, and Antioxidant Defense Confers Salinity Tolerance During Seed Germination in Wheat
Source: Plants (Basel). 2026 Jan 12;15(2):230. doi: 10.3390/plants15020230 (PMC12844750; doi:10.3390/plants15020230)
Supplement: Supplementary file 1 [file plants-15-00230-s001.zip › plants-3962194-supplementary.pptx]

## Slide 1
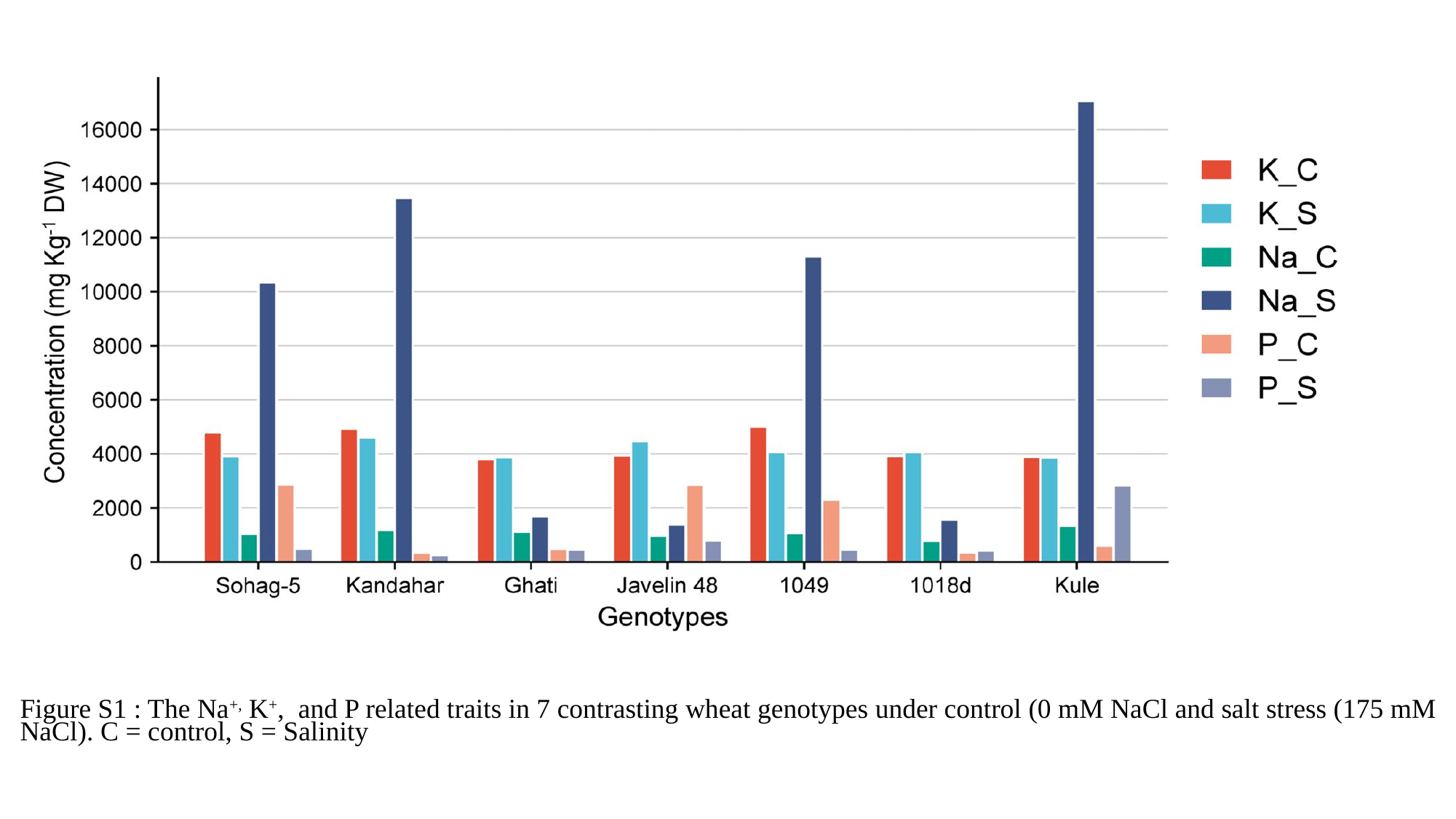

Figure S1 : The Na+, K+, and P related traits in 7 contrasting wheat genotypes under control (0 mM NaCl and salt stress (175 mM NaCl). C = control, S = Salinity

## Slide 2
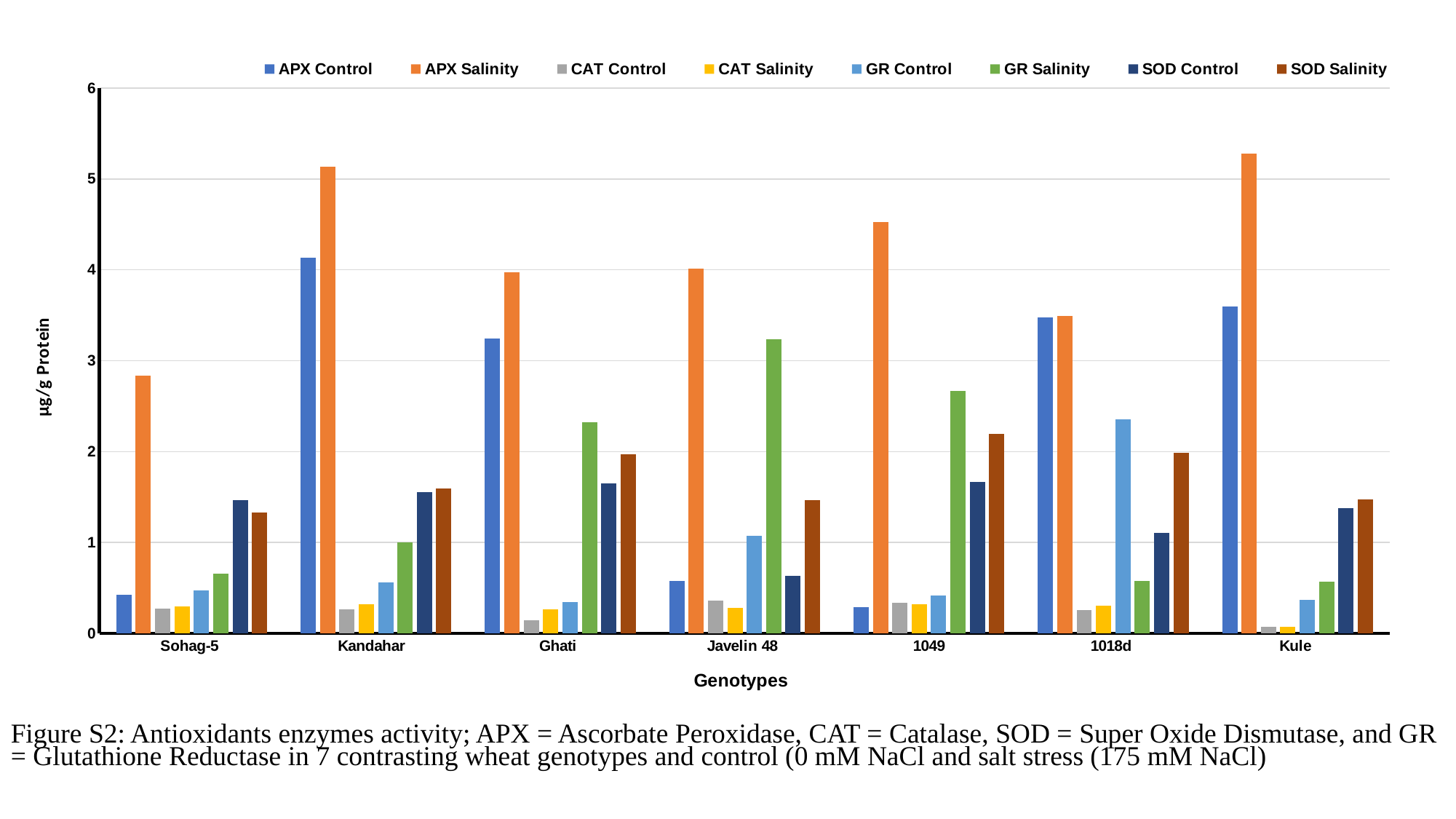

### Chart
| Category | APX | APX | CAT | CAT | GR | GR | SOD | SOD |
|---|---|---|---|---|---|---|---|---|
| Sohag-5 | 0.4266666666666667 | 2.8373333333333335 | 0.2748 | 0.29319999999999996 | 0.47120000000000006 | 0.6572 | 1.4660333333333335 | 1.3252666666666668 |
| Kandahar | 4.13 | 5.137999999999999 | 0.26066666666666666 | 0.32000000000000006 | 0.5579999999999999 | 0.9982000000000001 | 1.551866666666667 | 1.593066666666667 |
| Ghati | 3.2433333333333336 | 3.97 | 0.1406666666666667 | 0.268 | 0.34719999999999995 | 2.3249999999999997 | 1.6480000000000004 | 1.974166666666667 |
| Javelin 48 | 0.574 | 4.013333333333333 | 0.3565333333333333 | 0.2826666666666667 | 1.0726000000000002 | 3.2359999999999993 | 0.6351666666666668 | 1.4694666666666667 |
| 1049 | 0.2846666666666666 | 4.526666666666666 | 0.33640000000000003 | 0.3161333333333334 | 0.4154 | 2.666 | 1.6686000000000003 | 2.1973333333333334 |
| 1018d | 3.4766666666666666 | 3.4906666666666664 | 0.25453333333333333 | 0.3066666666666667 | 2.3560000000000003 | 0.5797 | 1.1021 | 1.9879000000000004 |
| Kule | 3.5933333333333333 | 5.278 | 0.07293333333333334 | 0.07466666666666667 | 0.37200000000000005 | 0.5703999999999999 | 1.3733333333333337 | 1.4729 |Figure S2: Antioxidants enzymes activity; APX = Ascorbate Peroxidase, CAT = Catalase, SOD = Super Oxide Dismutase, and GR = Glutathione Reductase in 7 contrasting wheat genotypes and control (0 mM NaCl and salt stress (175 mM NaCl)

## Slide 3
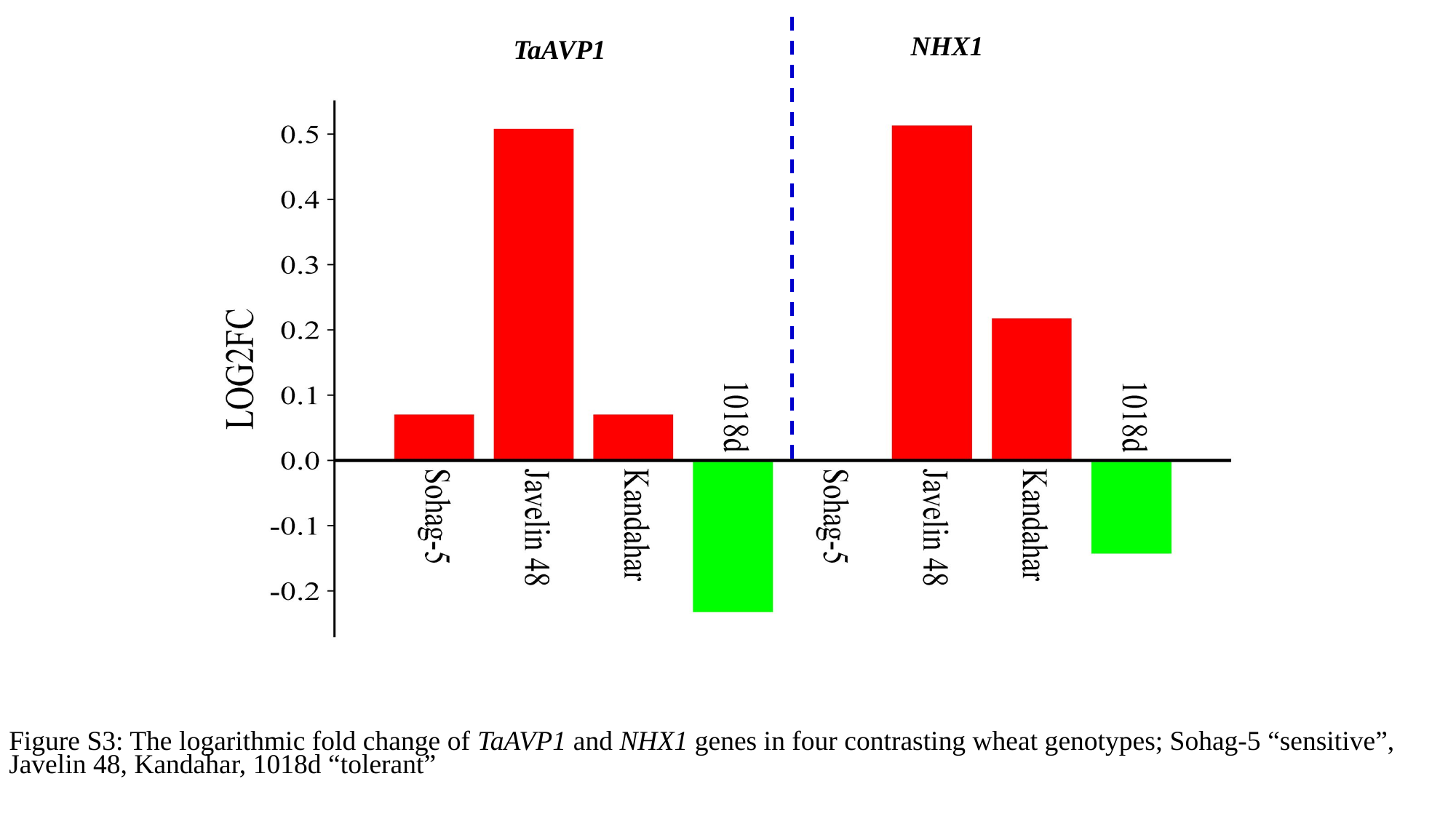

NHX1
TaAVP1
# Figure S3: The logarithmic fold change of TaAVP1 and NHX1 genes in four contrasting wheat genotypes; Sohag-5 “sensitive”, Javelin 48, Kandahar, 1018d “tolerant”
